# A Study of Absolute Pressure Inside the Cabins of Land Transport Vehicles—The Concept of a Ventilation System Regulating the Pressure in the Vehicle

**DOI:** 10.3390/s26020469

**Published:** 2026-01-10

**Authors:** Tomasz Janusz Teleszewski, Katarzyna Gładyszewska-Fiedoruk

**Affiliations:** 1 Department of HVAC Engineering, Faculty of Civil Engineering and Environmental Sciences, Bialystok University of Technology, Wiejska 45E, 15-351 Białystok, Poland; t.teleszewski@pb.edu.pl; 2 Institute of Environmental Engineering, Warsaw University of Life Sciences (SGGW), Nowoursynowska 166, 02-776 Warsaw, Poland

**Keywords:** absolute pressure, passenger car, bus ventilation system, train ventilation system

## Abstract

**Highlights:**

**What are the main findings?**

- Significant differences in absolute pressure occur during land travel (car, bus, train).
- A pressure change of 8 hPa within a 24 h period constitutes an unfavorable mechanical stimulus for the human body and causes changes in the excitability of the nervous system.

**What are the implications of the main findings?**

- Changes in absolute pressure undoubtedly cause changes in comfort and affect the human body; therefore, absolute pressure should be taken into account in assessing thermal comfort.
- It is worth implementing a pressure stabilization system in vehicles.

**Abstract:**

This paper presents the concepts of a vehicle pressure regulation ventilation system based on the results of absolute pressure measurements in land transport vehicles: passenger cars, buses and trains. Despite the fact that absolute pressure affects human well-being and health, this parameter is often overlooked in studies assessing thermal comfort. Absolute pressure measurements were taken during normal passenger transport operation. The studies were conducted for various terrain types: lowlands, highlands, and mountains. Absolute pressure fluctuations in land transport depended primarily on altitude, with the largest atmospheric pressure differences recorded in mountains and the smallest in lowlands. A pressure change of 8 hPa within a 24 h period constitutes an unfavorable mechanical stimulus for the human body and causes changes in the excitability of the nervous system. In all measurement series, absolute pressure fluctuations exceeded 8 hPa. Based on the results of absolute pressure measurements and altitude, a simplified model for predicting absolute pressure in transport vehicles was developed. To reduce absolute pressure fluctuations inside passenger land vehicle cabins, a ventilation scheme regulating pressure inside land vehicle cabins was proposed.

## 1. Introduction

The study and analysis of indoor microclimate parameters include issues related to the measurement of pollutants and monitoring of thermal comfort parameters [1,2], the design of measuring devices [3,4] and the regulation of ventilation systems [5]. One of the important microclimate parameters in rooms occupied by people is absolute pressure.

Absolute ambient pressure in rooms occupied by people affects well-being and health [6,7,8,9,10,11]. Publication [6] examined the effect of short-term weather parameters on knee joint pain. An increase in barometric pressure causes increased pain in patients with knee osteoarthritis. The relationship between blood pressure and atmospheric pressure in temperate climates at different times of the year was determined in [7]. The greatest effect of atmospheric pressure on blood pressure in temperate climates was observed during winter and spring [7].

Article [8] presented the results of research on the effect of atmospheric pressure on heart attacks and coronary heart disease. The lowest incidence of heart attacks occurred at an atmospheric pressure of 1016 mbar, while the highest incidence occurred at minimum and maximum values of atmospheric pressure [8].

Based on registered deaths between 2003 and 2011 in Guangzhou, China [9], a significant effect of minimum atmospheric pressure on the increase in mortality from cardiovascular causes was observed. Based on a study [10] of 250 patients with arterial hypertension aged 65–92, it was noted that the greatest complications occur at low pressures. Another health problem associated with pressure changes is non-epileptic seizures [11]. In the United States [11], a significant association was noted between atmospheric pressure and the occurrence of non-epileptic seizures. The increase in epileptic seizures by 31.8% and 11.1% occurred for atmospheric pressure that was lower or higher than the level generally considered comfortable (1013 hPa), respectively.

The pressure value in rooms where people stay depends primarily on the type of ventilation system [12,13,14,15] and the way the rooms are used [16,17]. In [18], it was concluded that the environmental pressure should be taken into account in the thermal comfort index in relation to the design of ventilation systems.

The popular indicator used to assess thermal comfort in enclosed spaces, PMV (Predicted Mean Vote) in ASHRAE-55 [19] and ISO7730 [20], is determined without taking into account the influence of atmospheric pressure. Atmospheric pressure is only included in psychrometric calculations. Atmospheric pressure has a direct impact on metabolic rate [18]. In car cabins, temperature, humidity, and carbon dioxide concentration are taken into account when assessing optimal air parameters [21].

The literature [22,23,24] provides general guidelines on the impact of atmospheric pressure differences over a given period on human health. Fluctuations in atmospheric pressure can significantly impact human health [22,25]. Optimal atmospheric pressure values for human health are 1013 hPa [11,26] and 1016 hPa [8].

In means of transport, the most common measurements were temperature [27,28], humidity [29,30], and carbon dioxide [31,32]. No pressure measurements were found in land passenger transport vehicles in the literature.

The aim of the work was to analyze the absolute pressure in selected types of land passenger transport vehicles: passenger cars, buses and trains, and to develop a concept of ventilation regulating the pressure in the passenger vehicle cabin with a simplified calculation model for determining the absolute pressure value as a function of altitude above sea level.

The following chapters present the remaining content of this article. Section 2 describes in detail the methodology for measuring absolute pressure, altitude above sea level, and vehicle travel routes. Section 3 presents the results and their discussion. A simplified model of absolute pressure in passenger vehicle cabins was constructed (Section 4). Additionally, a scheme for regulating and stabilizing pressure in a passenger car cabin was proposed. Section 5 contains the conclusions of this work.

## 2. Materials and Methods

Absolute pressure measurements were performed in a passenger car, bus and train. In the overland studies, the bus and train routes passed through lowlands, while the passenger car route passed through lowlands, uplands, and mountains. Lowlands are most often defined as areas below 200 m above sea level [33], uplands encompass altitudes ranging from 200 m to 300 m above sea level [34], and mountains can be located from 300 m above sea level, depending on the class [35].

**Figure 1:**
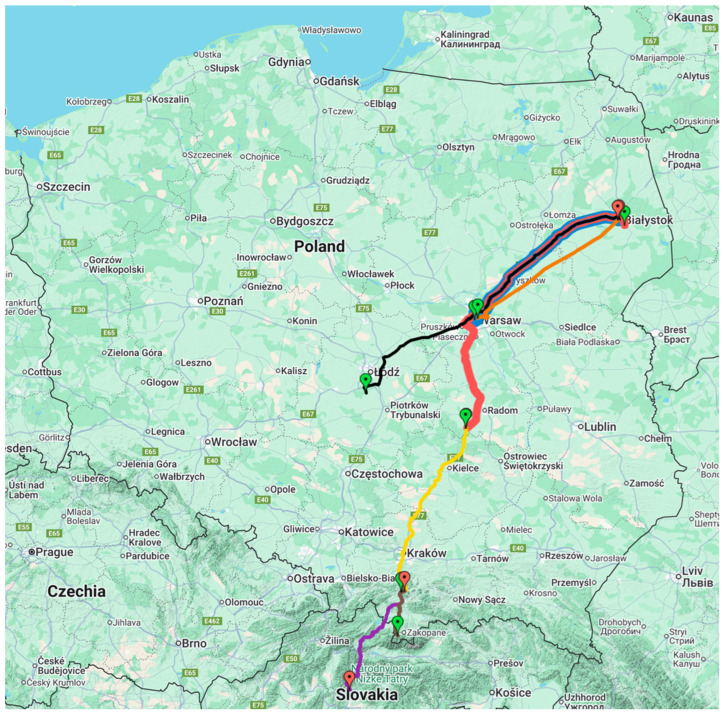
Location of the studied land routes on maps [36]: series: 1, 2, 8 (blue line); series: 3, 15 (red line); series 4, 7, 14 (yellow line); series: 5, 6 (purple line); series: 9 (black line); series: 10, 11, 12 (orange line); series:, 13 (brown line).

Table 1 presents the basic parameters of the 16 measurement series: measurement series number, beginning and end of the travel route, measurement date, vehicle type, travel duration, terrain type, and the name of the geographical region through which the route passed. Figure 1 show the travel routes on maps [36] (series no. 1–15). Passenger transport routes ran through lowlands (series 1–3 and series 8–12, 15), through uplands (measurement series 4, 7, 14) and mountains (series 5–6, 13).

A Testo data logger with an accuracy of ±3 hPa and a resolution of 0.1 hPa was used to measure absolute pressure. Prior to testing, the sensors were calibrated by an external calibration laboratory. To determine the exact measurement uncertainty of the Testo instrument, the absolute pressure measurement results of the Testo probe were compared with a mercury barometer with an accuracy of ±0.2 mm of mercury (0.27 hPa). The measurement uncertainty of the Testo probe was determined using the following formulas [37]:

(1) $$\delta p=\sqrt{{\left(B_{inst}\right)}^{2}+{\left(2\sigma_{run}\right)}^{2}},$$

(2) $$B_{inst}=p_{w}-\bar{p},$$

(3) $$\sigma_{run}=\sqrt{\frac{\sum_{i=1}^{n}{\left(\bar{p}-p_{i}\right)}^{2}}{n}},n=30$$

where *p_w_* is the atmospheric pressure value obtained from the mercury barometer, $\bar{p}$ is the arithmetic mean of the Testo atmospheric pressure measurements for 30 readings, and *σ_run_* is the standard deviation of the 30 readings.

The accuracy of the Testo probe determined according to formulas (1)–(3) was 1.1 hPa, while the relative error of the Testo probe determined as the difference in absolute pressure read from the mercury barometer and the Testo recorder related to the absolute pressure from the mercury barometer was 0.18%.

Altitudes above sea level were recorded using a Holux data logger with a horizontal accuracy of <2.2 m and a vertical accuracy of <5 m with a GPS (Global Positioning System) synchronization time of 0.1 ms.

## 3. Results and Discussion

**Figure 2:**
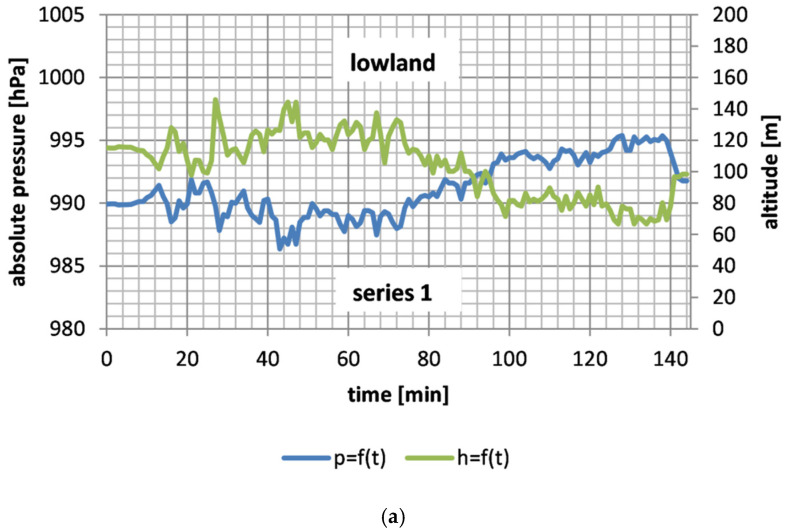

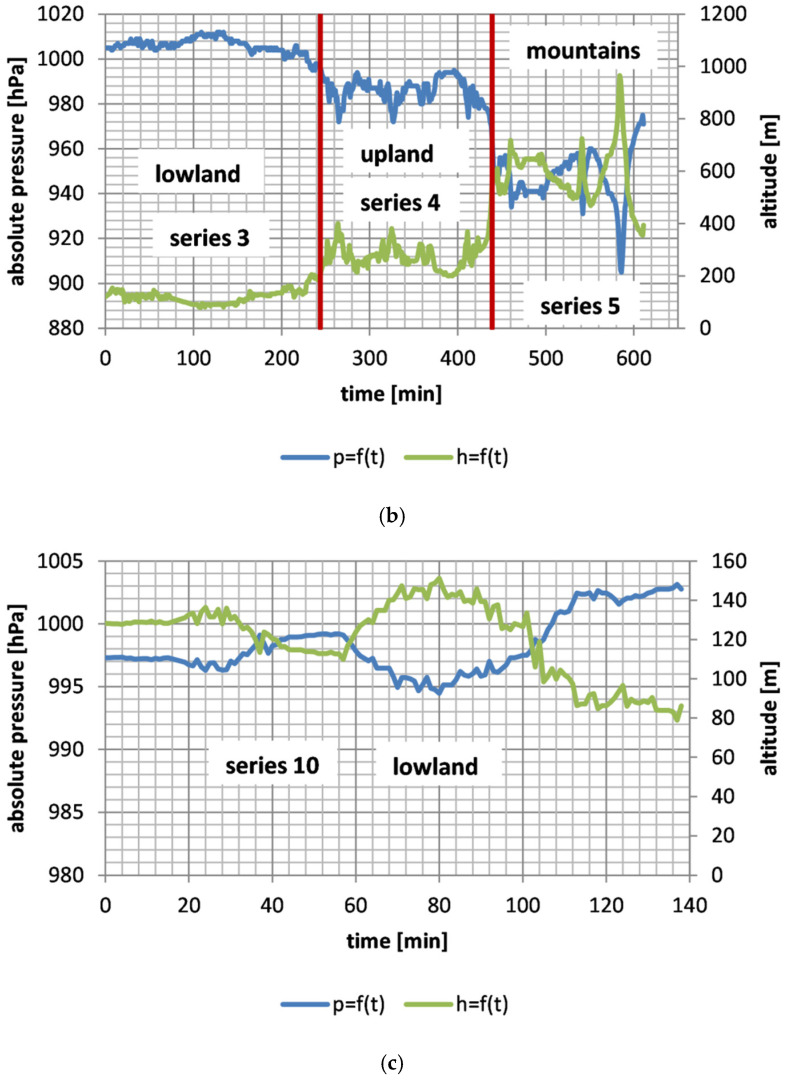
Dependence of absolute pressure and altitude as a function of time for selected measurement series of land transport: (**a**) series 1 (bus), (**b**) series 3–5 (passenger car), (**c**) series 10 (train).

Table 2 presents the minimum (*h_min_*), maximum (*h_max_*), mean (*h_avg_*) and standard deviation (*σh*) values of altitude above sea level, as well as the minimum (*p_min_*), maximum (*p_max_*), mean (*p_avg_*) and standard deviation (*σp*) values of absolute pressure for 15 measurement series. Additionally, Table 2 presents the differences in altitude above sea level between the maximum and minimum values (Δ*h* = *h_max_* − *h_min_*) and the differences in absolute pressure between the maximum and minimum values (Δ*p* = *p_max_* − *p_min_*). Figure 2a–c present changes in altitude above sea level and absolute pressure as a function of time, for example, measurement series of land transport (series 1, 3, 4, 5 and 10, respectively).

The absolute pressure values in passenger car, bus and train cabins depended primarily on the altitude above sea level (Figure 2a–c), which is consistent with the classical theory [38]. With increasing height above sea level, the absolute pressure decreases, which is caused by the lower pressure of the air column at the measuring point [38]. The trends of absolute pressure changes during the passenger car, bus and train cabin are symmetrical to the trends of changes in altitude above sea level (Figure 2a–c), which indicates a small impact of ventilation operation on absolute pressure in these vehicles.

**Figure 3:**
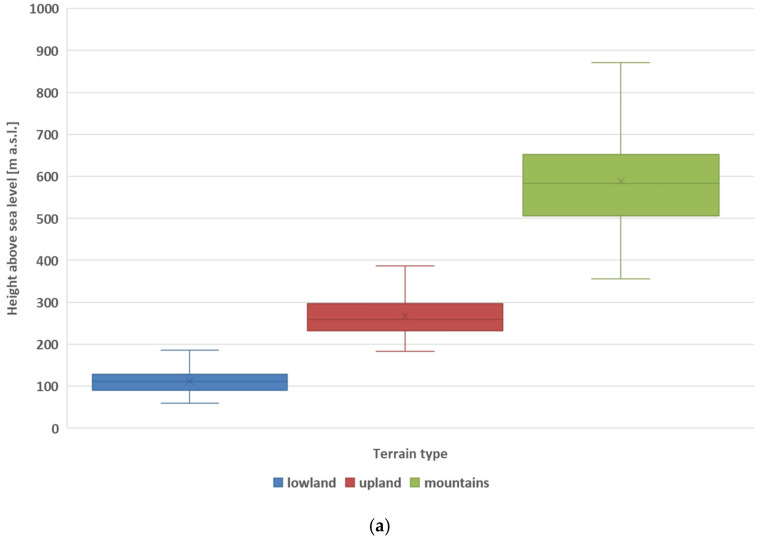

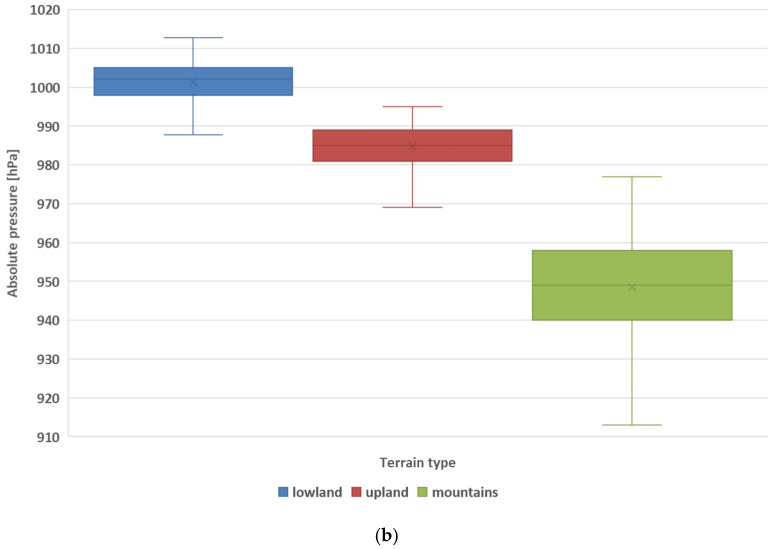
Box plot for altitude above sea level (**a**) and absolute pressures in the passenger vehicle cabin (**b**).

Figure 3a,b present box plots for altitude above sea level and absolute pressure in passenger vehicle cabins, respectively, for various terrain types. The lowest absolute pressure values occur in mountainous areas (Figure 3b), which is related to the higher altitude above sea level in the mountains (Figure 3a) compared to other landforms. The greatest fluctuations in absolute pressure occur in the mountains (Figure 3b), which is caused by significant changes in altitude above sea level in these areas (Figure 3a). Special attention should be paid to safety when driving in mountainous terrain, taking into account the impact of significant changes in absolute pressure on the driver and passengers.

**Figure 4:**
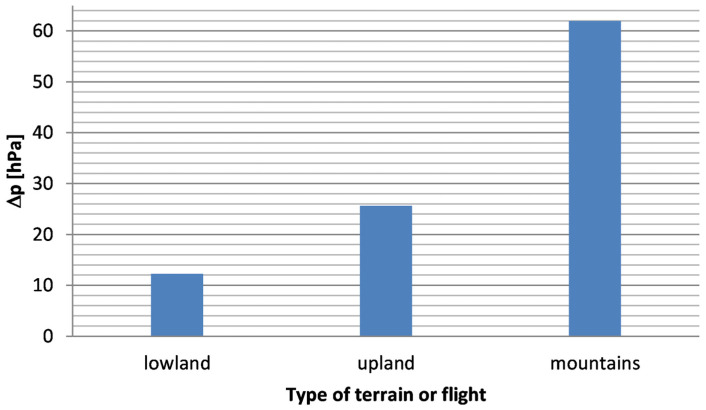
Average maximum absolute pressure differences during travel for lowland areas, uplands and mountains.

Figure 4 presents the average maximum absolute pressure differences inside passenger transport vehicles during travel for lowland areas (series 1–3, 8–12, 15), uplands (series 4, 7, 14) and mountains (series 5–6, 13) The largest pressure differences in land transport occur in mountainous areas and highlands, while the smallest absolute pressure differences occur in lowland areas. In the case of travel through the mountains, the average value of absolute pressure differences (series 5–6, 13) is as much as 5.1 times greater than the average value of absolute pressure differences for land transport through the lowlands (series 1–3, 8–12, 15). A pressure change of 8 hPa within a 24 h period constitutes an unfavorable mechanical stimulus for the human body and causes changes in the excitability of the nervous system [22]. In all measurement series, absolute pressure fluctuations exceeded 8 hPa. Daily fluctuations in atmospheric pressure above 5.5 hPa may contribute to an increased number of epileptic events for people who are susceptible to these diseases [25].

Sudden and significant changes in atmospheric pressure cause compression and expansion of air in the middle ear, which may cause discomfort in hearing [21,22]. Boksha and Boguckij [23] developed a scale of sensations of atmospheric pressure changes depending on the absolute pressure difference. The scale of sensations [21,22] is based on the following pressure difference ranges: within the range up to 4.0 hPa, weak changes in sensations due to pressure are assumed, then for the Δ*p* range from 4.1 hPa to 8.0 hPa, moderate changes, for the Δ*p* range from 4.1 hPa to 8.0 hPa, moderate changes, for the Δ*p* range from 8.1 hPa to 12.0 hPa, strong changes, and for the Δ*p* range above 12.0 hPa, very strong changes. Taking into account the above scale of changes in sensations due to differences in atmospheric pressure, it can be stated that in the case of lowlands there is a full range of sensations, while in the case of highlands and mountains there are very strong sensations.

**Figure 5:**
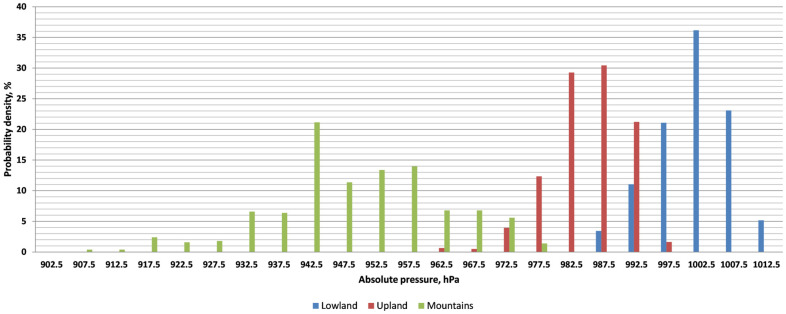
Histogram of absolute pressures depending on the type of terrain for measurement series 1–15.

Based on the absolute pressure measurements for the series, a histogram of the frequency of absolute pressure intervals was plotted (Figure 5) for journeys through lowlands, uplands, and mountains. The frequency distribution of a given absolute pressure was determined by summing the number of one-minute absolute pressure measurements in a given pressure interval [*p_i_*, *p_i_*_+1_):

(4) $$\mathrm{Probability}\;\mathrm{density}=\frac{n_{i}}{N}\times 100\%,$$

where *n_i_*—number of measurements from a given interval [*p_i_*, *p_i_*_+1_), and *N* is the number of all measurements in a given period.

Absolute pressures were divided into 5 hPa numerical intervals with range boundaries ranging from 900 to 905 hPa to 1010–1015 hPa. Figure 5 shows the middle values of the adopted ranges on the horizontal axis, while the vertical axis shows the frequency of a given pressure, expressed as a percentage. In the lowlands, the highest probability of 36.2% occurs for the pressure range [1000 Pa, 1005 Pa]. In the uplands, the pressure ranges [980 Pa, 985 Pa) and [985 Pa, 990 Pa) occurred most frequently, with probabilities of 29.3% and 30.4%, respectively. In the mountains, the dominant pressure range was [940 Pa, 945 Pa) with a probability of 21.2%. The optimal pressure range [1010 Pa, 1015 Pa) [8,11,26] appears with a probability of 5.2% when traveling through lowlands.

Analyzing the data from [8], it can be concluded that the lowest probability of myocardial infarction may occur in lowland areas. A low drop in atmospheric pressure below 1013 hPa, associated with blood pressure, is associated with a greater number of cardiovascular and cerebral complications [10]. On the other hand, the highest mortality [9] occurs at extremely high atmospheric pressures above 1020 hPa, although such high absolute pressures were not recorded in this study. Pressure lower than comfort pressure (1013 kPa) may also cause epileptic seizures [11] in people treated for these conditions.

## 4. Simplified Model of Absolute Pressure as a Function of Altitude

**Figure 6:**
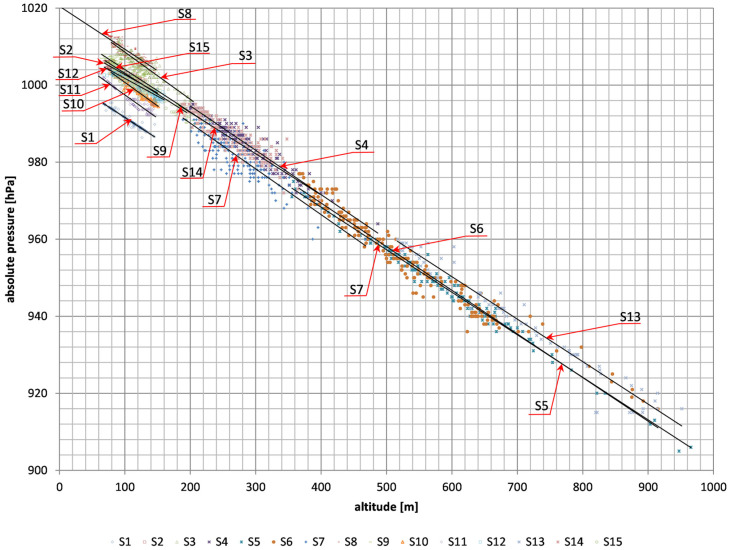
Dependence of absolute pressure as a function of altitude for measurement series 1–15.

Figure 6 shows the dependencies of absolute pressure inside the passenger car, bus and train cabin on altitude above sea level for measurement series 1–15. For low altitudes up to 1500 m, the change in absolute pressure as a function of altitude can be described by a simplified relationship in which the pressure drops by approximately 11 hPa for each 100 m increase in altitude at a pressure above sea level of 1013.2 hPa [39].

Based on the measurements of absolute pressure and altitude above sea level, a model based on linear regression was determined:

(5) $$p_{abs}=b-a\cdot h,$$

where the coefficient *a* is the change in the absolute pressure value in hPa per 1 m of height, while the coefficient *b* is the equivalent of the absolute pressure above sea level.

The coefficients *a* and *b* depend on local conditions related to air parameters and the operation of ventilation systems in means of transport. The coefficients *a* and *b* are presented in Table 2. In the case of land transport, the average value of the coefficient *a* from 15 measurement series is 0.112 hPa, while the average value of the coefficient *b* is 1013.6 hPa, and these values are close to the value of the absolute pressure of outside air [39].

**Figure 7:**
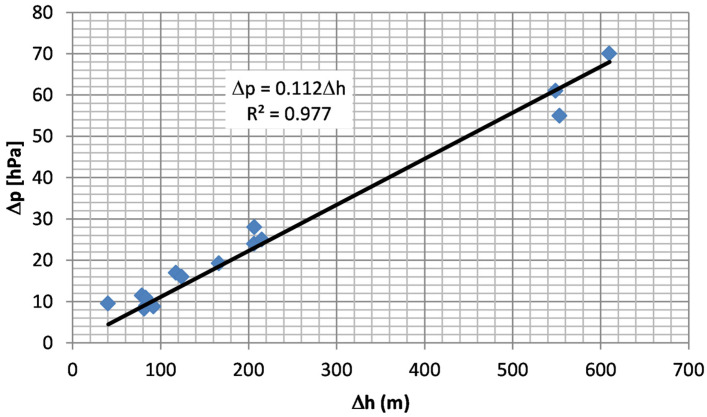
Dependence of extreme differences in absolute pressure as a function of extreme differences in height above sea level based on measurement series 1–15.

Figure 7 shows the dependence of extreme differences between the maximum (p_max_) and minimum (*p_min_*) absolute pressure on the extreme differences in height above sea level (*h_max_* − *h_min_*) for measurement series no. 1–15 (Table 2). Based on the determined linear regression (Figure 6), it can be concluded that a 1 m height change generates a pressure change of 0.112 hPa, which is also similar to the outdoor air parameters [39].

**Figure 8:**
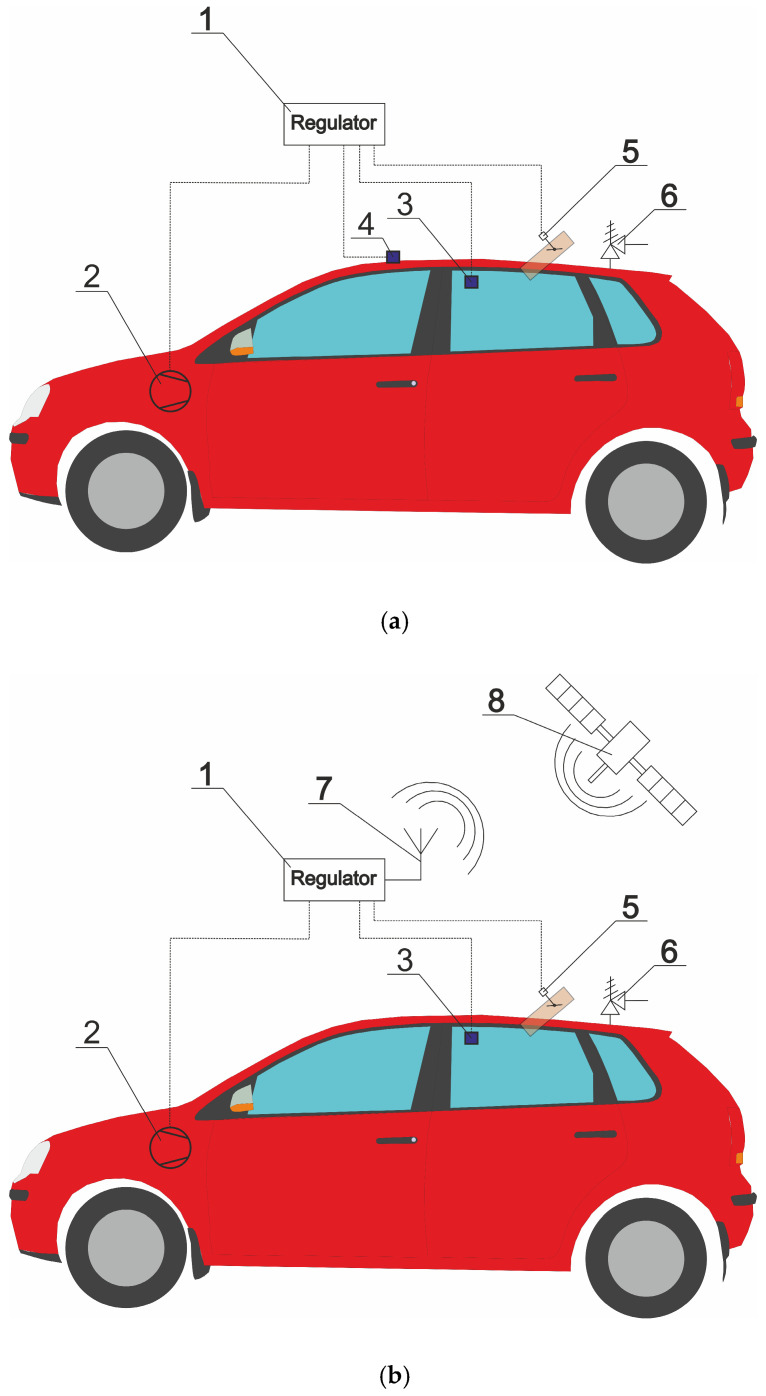
Simplified diagram of pressure regulation in the car cabin in the variant without (**a**) and with GPS (**b**): 1—regulator, 2—compressor/vacuum pump, 3—internal pressure sensor, 4—external pressure sensor, 5—regulating valve, 6—safety valve, 7—antenna, 8—GPS satellite.

Due to the fact that absolute pressure fluctuations affect human comfort, well-being and health [8,11,22,23,24], it is worth implementing a pressure stabilization system in vehicles. Figure 8a,b show proposed pressure regulation schemes for a passenger car cabin. The regulator (1) should be set to the desired pressure inside the car cabin. The recommended pressure value is 1013 hPa, which is optimal for oxygen saturation and proper functioning of the human body [8,11,26]. The pressure inside the cabin is read using a pressure sensor (3), which can be placed on the car’s ceiling. An air compressor (2) with the function of generating both overpressure and underpressure, and a control valve (5), maintain the appropriate pressure inside the cabin. In a combustion engine vehicle, a compressor driven by a V-belt can be used, while in an electric vehicle, a compressor driven by an electric motor can be used.

Pressure regulation can be implemented in two variants. In the first variant (Figure 8a), the pressure inside the cabin is set as a constant value on the controller (1) and is maintained regardless of the vehicle’s altitude above sea level. To adjust the compressor’s output (2), it is also necessary to measure the pressure outside the vehicle using an external pressure sensor (3). Regulating the pressure inside the cabin using the compressor (2) can be achieved, for example, by changing the compressor rotor speed as a function of the pressure difference between sensors (3) and (4), which is an energy-saving solution. Reducing excess pressure can be achieved by opening the pressure relief valve (5). The cabin pressure regulation system is protected by a safety valve (6), which prevents excessively low or high pressure inside the vehicle in the event of a system failure.

In the second variant (Figure 8b), cabin pressure regulation can be supported by a planned route in the navigation system connected to the controller (1), which connects via an antenna (7) to a satellite (8) to read GPS points. Based on the planned route and current GPS altitude readings, pressure changes due to changes in terrain elevation can be predicted. In the second variant, the external pressure is determined from Equation (5) based on altitude above sea level. The presented solution for stabilizing pressure in a passenger car cabin (Figure 8a,b) can also be used in buses and trains. Control of the operation of the ventilation system with pressure regulation can be implemented within the Internet of Things (IoT) [40].

### Limitations and Future Research

Absolute pressure is most often overlooked in studies on thermal comfort and air quality in occupied spaces, which is why this topic requires more extensive research. In addition to altitude, external weather conditions, vehicle cabin ventilation, and vehicle operation also influence absolute pressure in land transport cabins. A broader understanding of changes in absolute pressure in vehicle cabins requires additional research. The main limitations of this publication, which will also serve as the basis for further research and analysis, are presented below:

1. This publication proposes a preliminary pressure regulation scheme in a vehicle without selecting individual devices. Further research is planned to involve building a prototype of a ventilation system with pressure regulation inside the car and carrying out tests.
2. The rate of pressure change in the vehicle cabin was not analyzed, which would be necessary to propose a detailed procedure for regulating and monitoring changes in absolute pressure inside and outside the vehicle. Analyzing pressure rate changes requires the use of shorter time intervals; therefore, this topic will be explored in subsequent studies.
3. Including atmospheric pressure in the PMV (Predicted Mean Vote) thermal comfort index primarily requires survey research, which will be conducted in subsequent studies.
4. The external air parameters during driving and the impact of changing atmospheric conditions on the absolute pressure inside the vehicle were not analyzed.
5. The paper also does not analyze vehicle operating conditions such as constant or variable vehicle speed, the impact of window and door opening on absolute pressure in the vehicle cabin, or the impact of ventilation type and operation on absolute pressure in the vehicle cabin.

## 5. Conclusions

Significant differences in absolute pressure occur during land travel (car, bus, train). In the case of land transport, absolute pressure depends primarily on altitude. The largest fluctuations in absolute pressure for land transport were recorded in the mountains and highlands, while the smallest fluctuations were recorded in the lowlands. The obtained results confirm the close dependence of absolute pressure on height above sea level, which is approximately 0.11 hPa/m for land transport. Changes in absolute pressure undoubtedly cause changes in comfort and affect the human body; therefore, absolute pressure should be taken into account in assessing thermal comfort, just as it is with temperature and humidity. A way to reduce absolute pressure is to use a ventilation system to maintain a constant pressure inside the passenger vehicle cabin.

Practical implications:

1. The obtained results may find application in the design of HVAC and ventilation systems in passenger cars, enabling the regulation of cabin pressure, as well as temperature and humidity control.
2. The simplified pressure model proposed in this study can support the prediction of pressure variations along different routes and assist in adaptive control of cabin climate systems.
3. The proposed pressure control system based on a pressure sensor, compressor and GPS data can be used to stabilize pressure in land transport vehicles, improving passenger comfort, especially in areas with significant height differences above sea level.
4. The findings may contribute to the development of comfort assessment standards (such as ASHRAE 55 or ISO 7730) by including absolute pressure as a factor influencing human well-being.
5. The results can also be used in transport planning and personnel training, particularly for managing the comfort of passengers sensitive to pressure fluctuations (e.g., elderly individuals or those with cardiovascular diseases).

## Figures and Tables

**Table 1:** Basic parameters of measurement series 1–15.

No. Series	Land or Air Route	Date	Vehicle/Aircraft Type	Travel Time	Terrain Type	The Name of the Land
-	-	-	-	min	-	
1	Bialystok-Warsaw	12.04.2023	Bus	144	Lowland	North Podlasie Lowland, Masovian Lowland
2	Warsaw-Bialystok	20.04.2023	Bus	165	Lowland	North Podlasie Lowland, Masovian Lowland
3	Bialystok-Szydlowiec	01.05.2023	Passenger car	244	Lowland	North Podlasie Lowland, Masovian Lowland
4	Szydlowiec-Krzeczow	01.05.2023	Passenger car	195	Upland	Przedborska Upland, Kraków-Częstochowa Upland, Silesian Upland
5	Krzeczow-Banská Bystrica	01.05.2023	Passenger car	172	Mountains	Carpathians
6	Banská Bystrica-Krzeczow	05.05.2023	Passenger car	233	Mountains	Carpathians
7	Krzeczów- Szydłowiec	05.05.2023	Passenger car	213	Upland	Przedborska Upland, Kraków-Częstochowa Upland, Silesian Upland
8	Warsaw-Bialystok	18.05.2023	Bus	182	Lowland	North Podlasie Lowland, Masovian Lowland
9	Dłutów-Bialystok	26.05.2023	Passenger car	254	Lowland	North Podlasie Lowland, Masovian Lowland
10	Bialystok-Warsaw	17.05.2023	Train	138	Lowland	North Podlasie Lowland, Masovian Lowland
11	Warsaw-Białystok	01.06.2023	Train	146	Lowland	North Podlasie Lowland, Masovian Lowland
12	Bialystok-Warsaw	13.06.2023	Train	159	Lowland	North Podlasie Lowland, Masovian Lowland
13	Kiry-Krzeczow	10.08.2024	Passenger car	97	Mountains	Carpathians
14	Krzeczow-Szydlowiec	10.08.2024	Passenger car	200	Upland	Przedborska Upland, Kraków-Częstochowa Upland, Silesian Upland
15	Szydlowiec-Bialystok	10.08.2024	Passenger car	296	Lowland	North Podlasie Lowland, Masovian Lowland

**Table 2:** Minimum, maximum values, average, standard deviation, differences between the maximum and minimum values of measured altitudes above sea level and absolute pressure, and coefficients of Formula (1) for measurement series 1–15.

No. Series		Height Above Sea Level					Absolute Pressure				Model Coefficients and Coefficient of Determination (5)		
	*h_min_*	*h_max_*	*h_avg_*	*σh*	Δ*h*	*p_min_*	*p_max_*	*p_avg_*	*σp*	Δ*p*	*a*	*b*	*R* ^2^
-	m a.s.l.	m a.s.l.	m a.s.l.	m a.s.l.	m	hPa	hPa	hPa	hPa	hPa	hPa	hPa	-
1	62.0	145.8	105.8	20.3	83.8	986.3	995.4	991.2	2.3	9.0	0.110	1002.6	0.947
2	68.5	150.0	103.5	19.7	81.5	998.1	1006.3	1002.4	2.0	8.2	0.096	1012.3	0.841
3	87.3	204.8	132.5	26.9	117.6	995.0	1012.0	1005.9	3.4	17.0	0.123	1020.8	0.876
4	200.0	486.8	279.9	44.9	286.8	970.0	996.0	986.0	5.4	26.0	0.115	1017.6	0.923
5	355.5	965.5	577.6	108.0	610.1	905.0	975.0	947.9	11.6	70.0	0.110	1012.4	0.989
6	366.1	915.1	529.6	111.2	549.0	916.0	977.0	953.1	12.9	61.0	0.113	1014.7	0.954
7	188.9	468.2	270.3	44.5	279.3	963.0	991.0	982.1	6.2	28.0	0.119	1013.9	0.810
8	74.6	115.0	87.3	7.4	40.4	1003.3	1012.8	1008.2	2.5	9.5	0.112	1020.5	0.807
9	64.3	230.7	117.8	41.0	166.3	988.7	1008.0	1002.5	4.6	19.3	0.110	1015.0	0.909
10	73.1	152.2	119.6	19.5	79.1	994.5	1006.0	1000.6	3.3	11.5	0.123	1013.1	0.865
11	70.4	153.6	113.0	19.2	83.3	992.2	1003.1	997.2	3.2	10.9	0.121	1009.5	0.929
12	68.3	160.3	114.9	26.4	92.0	995.7	1004.5	1000.3	2.6	8.8	0.095	1011.1	0.963
13	398.0	951.5	701.8	125.0	553.6	915.0	970.0	938.8	13.6	55.0	0.110	1016.4	0.960
14	182.7	398.0	258.4	45.1	215.2	970.0	995.0	986.6	5.3	25.0	0.107	1014.3	0.885
15	70.0	194.2	113.6	26.8	124.2	991.0	1007.0	1001.6	3.1	16.0	0.108	1013.8	0.853

## Data Availability

The data generated and analyzed during this study are available on request from the corresponding author. The data are not publicly available due to ongoing research and analysis.

## Nomenclature

*a*	coefficient of Equation (1) in (hPa/m)
*b*	coefficient of Equation (1) in (hPa)
*h*	height above sea level (m a.s.l.)
*h_avg_*	average value of altitude above sea level (m a.s.l.)
*h_min_*	minimum value of altitude above sea level (m a.s.l.)
*h_max_*	maximum value of altitude above sea level (m a.s.l.)
*n_i_*	number of measurements from a given interval [*p_i_*, *p*_*i*+1_) according to Formula (4)
*N*	number of all measurements in a given period according to Formula (4)
*p*	absolute pressure (hPa)
*p_avg_*	mean absolute pressure (hPa)
*p_min_*	minimum absolute pressure value (hPa)
*p_max_*	maximum absolute pressure value (hPa)
Greek symbols	
*δp*	uncertainty of the absolute pressure measuring probe determined according to Formula (1)
Δ	difference
*σh*	standard deviation of altitude above sea level (m a.s.l.)
*σp*	standard deviation of absolute pressure in vehicle cabins (hPa)
